# Nodular anterior scleritis associated with Berger’s disease

**DOI:** 10.5935/0004-2749.20210011

**Published:** 2025-02-02

**Authors:** Emanuelly Giovaneli Constancio, Débora Letícia Souza Alves, Luiz Guilherme Marchesi Mello, Lídia Balarini da Silva, Fábio Petersen Saraiva, Júlia Polido, Thiago Cabral

**Affiliations:** 1 Department of Specialized Medicine, Universidade Federal do Espírito Santo, Vitória, ES, Brazil; 2 Division of Ophthalmology, Universidade de São Paulo, São Paulo, SP, Brazil; 3 Rheumatology Service, Universidade Federal do Espírito Santo, Vitória, ES, Brazil; 4 Vision Center Unit, Ophthalmology Department, Universidade Federal do Espírito Santo, Vitória, ES, Brazil; 5 Department of Ophthalmology, Universidade Federal de São Paulo, São Paulo, SP, Brazil

**Keywords:** Glomerulonephritis, Immunoglobulin A, Scleritis, Azathioprine, Cyclophosphamide, Case report, Glomerulonefrite, Imunoglobulina, Esclerite, Azatioprina, Ciclofosfamida, Relatos de casos

## Abstract

A 45-year-old female patient presented with a complaint of right eye redness and pain for 7 days. She was under investigation for urinary abnormalities and reported a previous history of recurrent oral ulcers and ocular hyperemia in both eyes. Best-corrected visual acuity was 20/30 and 20/20 in the right and left eyes, respectively. Slit-lamp biomicroscopy of the ocular surface of the right eye revealed nasal scleral hyperemia that persisted after instillation of topical phenylephrine 10%, reinforcing the diagnosis of anterior scleritis. Renal biopsy showed immunoglobulin A immune complexes and confirmed the suspected diagnosis of Berger’s disease. Maintenance immunosuppressive therapy with azathioprine following a 6-month induction of remission with cyclophosphamide was necessary after pulse therapy with methylprednisolone. Scleritis is usually related to systemic autoimmune diseases, such as rheumatoid arthritis, and polyangiitis. Herein, we describe a rare case of unilateral anterior scleritis associated with Berger’s disease.

## Abstract (pt)

Paciente de 45 anos, sexo feminino queixava-se de hiperemia e dor no olho direito há sete dias. Encontrava-se sob investigação de alterações urinárias e relatou história pregressa de úlceras orais e hiperemia ocular bilateral recorrentes. A acuidade visual corrigida era de 20/30 no olho direito e 20/20 no esquerdo. A biomicroscopia da superfície ocular do olho direito revelou intensa hiperemia escleral em região nasal que persistiu após a instilação de fenilefrina tópica a 10%, reforçando o diagnóstico clínico de esclerite anterior unilateral. A biópsia renal revelou a presença de imunocomplexos de IgA e confirmou a hipótese de doença de Berger. Uma terapia imunossupressora de manutenção com azatioprina após 6 meses de indução de remissão com ciclofosfamida foi necessária após pulsoterapia com metilprednisolona. A esclerite geralmente está relacionada a doenças autoimunes sistêmicas, como artrite reumatoide e poliangeite. Descrevemos aqui um caso raro de esclerite anterior unilateral associada à doença de Berger.

## INTRODUCTION

Immunoglobulin A nephropathy (IgAN), also termed Berger’s disease, is one of the most common primary glomerulopathies worldwide. This immune complexmediated disease typically affects males, in the second and third decades of life, and may be asymptomatic or manifest with hematuria and/or proteinuria. Renal biopsy is essential for its diagnosis, taking into account that IgA deposits may be observed even in patients without evidence of kidney disease. Systemic manifestations may frequently include arterial hypertension and chronic renal failure in the late stages of the disease, although it rarely affects the eye^(1)^.

Scleritis is an immune-mediated lesion characterized by painful inflammation of the sclera^(2)^. Although it can occur independently, up to 50% of patients present an underlying disease, such as connective tissue disorders, infectious agents, or previous history of trauma^(2,3)^. Rheumatoid arthritis, granulomatous polyangiitis, and systemic lupus erythematosus are the most common systemic disorders associated with scleritis. Commonly reported infectious agents include *Mycobacterium tuberculosis*, varicella-zoster virus, *Treponema pallidum*, and *Borrelia burgdorferi*^(3,4)^.

Ocular involvement in IgAN is uncommon, and few reports described its association with anterior scleritis^(5-9)^. Herein, we describe a rare case of unilateral nodular anterior scleritis in a patient with IgAN and highlight the importance of routine urinary laboratory investigation in patients with scleral inflammation.

## CASE REPORT

A 45-year-old female complained of redness and ocular pain in right eye for 7 days. She reported previous episodes of ocular hyperemia in both eyes, recurrent oral ulcers, systemic arterial hypertension, non-nephrotic proteinuria, hematuria without erythrocyte dysmorphism and normal renal function (under investigation by the Rheumatology and Nephrology Service).

**Figure 1:**
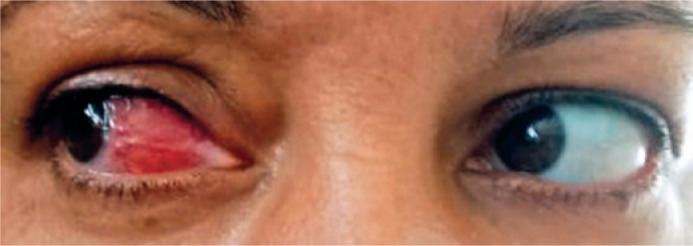
Image of both eyes showing nodular anterior scleritis in the nasal region of the right eye.

On ocular examination, best-corrected visual acuity was 20/30 in the right eye (OD) and 20/20 in the left eye. Pupillary reactions, slit-lamp biomicroscopy of the anterior segment, intraocular pressure, and fundus examination were normal. Slit-lamp biomicroscopy of the ocular surface revealed intense nasal scleral hyperemia (Figure 1) that persisted after instillation of topical phenylephrine 10%, which, together with the painful eye, confirmed our diagnosis of unilateral anterior nodular scleritis. Owing to its hypothesized association with Behçet’s disease, spondyloarthritis, systemic lupus erythematosus, or IgAN, pulse therapy with methylprednisolone (1 g/day for 3 days) followed by an oral corticosteroid-tapering regimen was prescribed after ruling out the presence of infectious diseases.

**Figure 2:**
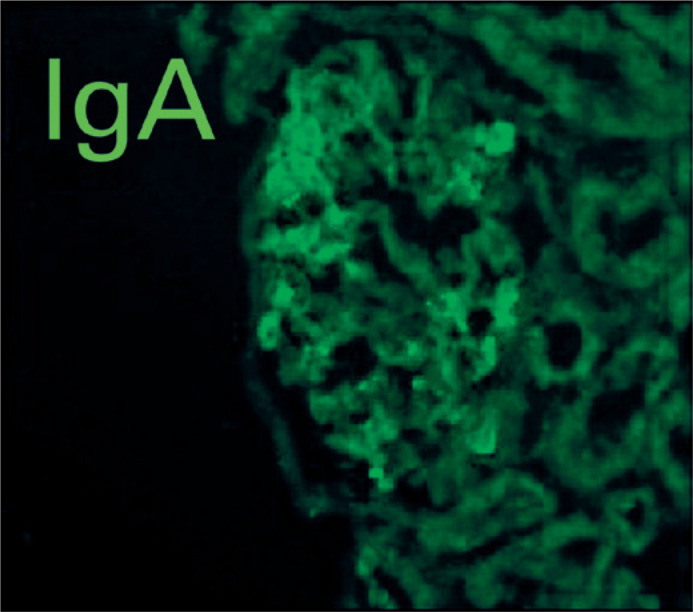
Renal biopsy with immunofluorescence staining showing granular deposition of immunoglobulin A (IgA) in a mesangial pattern with low intensity (1 of 3 points).

Laboratory tests revealed the following: a normal complete blood count, serum creatinine, blood urea nitrogen, C-reactive protein, erythrocyte sedimentation rate, and complement levels; negative antinuclear antibodies, anti-double stranded DNA, anti-Smith, anti-ribo nucleoprotein, anti-human leukocyte antigen-B27, anti-human immunodeficiency virus-1 and -2, Venereal Disease Research Laboratory test, and purified protein derivative test; negative anti-Toxoplasma IgM and positive IgG. A 24-h urine analysis revealed non-nephrotic proteinuria, urinary casts, and hematuria without dysmorphic erythrocytes. Finally, renal biopsy showed mild and focal mesangial proliferation and expansion, glomerular synechiae, and normal vessels, without atrophy or fibrosis. Immunofluorescence evidenced granular deposits of IgA in a mesangial pattern with low intensity and confirmed the diagnosis of IgAN (Figure 2).

**Figure 3:**
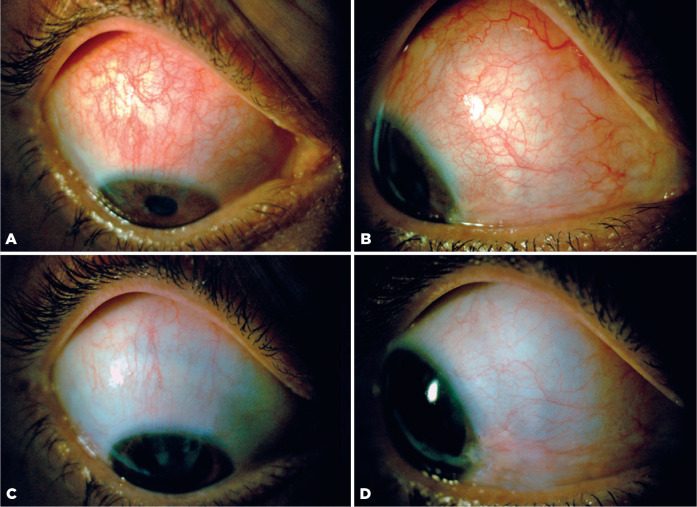
(A, B) Slit-lamp biomicroscopy of the right eye showing superior and nasal scleritis 3 days after pulse therapy with methylprednisolone. (C, D) At 40 days after pulse therapy, with complete improvement of nasal and superior inflammation of the right eye.

There was no significant improvement of the scleritis immediately after three consecutive daily intravenous methylprednisolone pulses of 1 g (Figures 3A, 3B). However, 40 days after pulse therapy, the best-corrected visual acuity improved to 20/20 in both eyes and the scleral inflammation completely resolved without sequelae (Figures 3C, 3D). Immunosuppression with cyclophosphamide (0.75 g/m^2^ of body surface area/month) for 6 months and maintenance treatment with azathioprine was initiated due to the severity of the disease, the inherent risk of new episodes of scleritis, and the late response to pulse therapy with methylprednisolone. At 14 months of follow-up, the patient did not show recurrence after therapy with azathioprine.

## DISCUSSION

Berger’s disease (IgAN) is the most frequent occurring primary glomerulonephritis^(1)^. However, the disease is usually asymptomatic in the early phases. In most cases, IgAN is restricted to the kidney and rarely affects the eye. A large proportion of patients with scleritis present an underlying disease^(2,3)^. Therefore, systemic investigation of connective tissue disorders and infectious disease is mandatory. In our case, the presence of scleritis in a patient with recurrent oral ulcers and normal renal function associated with non-nephrotic proteinuria led us to the differential diagnoses of Behçet’s disease, spondylarthritis, and systemic lupus erythematosus.

Ocular involvement in IgAN is uncommon. A systematic literature review showed episcleritis as the main ocular manifestation of IgAN^(4)^ and the association of Berger’s disease with anterior scleritis has been rarely described^(5-9)^. In this case, laboratory examinations ruled out numerous infectious and non-infectious causes of anterior scleritis. However, urinalysis played a pivotal role in guiding the etiological investigation of glomerular disease, due to the presence of non-nephrotic proteinuria, urinary casts, and hematuria without dysmorphic erythrocytes. The immunofluorescent evaluation of renal biopsy was decisive to reach a definitive diagnosis. In a patient with scleritis, IgAN should be considered even in the absence of urinary symptoms^(8)^.

Complement activation through alternative and lectin pathways plays a key role in the pathogenesis of IgAN, leading to systemic circulation of immune complexes and locally in the kidneys^(10)^. The exact pathophysiology of the relationship between IgAN and scleritis is uncertain^(4)^. An episcleral biopsy in a patient with IgAN and episcleritis revealed dimeric IgA-secreting plasma cells, suggesting that ocular surface immunity may be involved in ocular manifestations in this nephropathy^(11)^. Ocular IgA may also be related to the development of scleritis in IgAN. However, further investigations are warranted.

Systemic medications for the treatment of scleritis are frequently required to reduce inflammation and ocular damage. Corticosteroids with or without other immunosuppressive drugs should be considered in patients with associated autoimmune disease^(12)^. Thus far, there is no optimal treatment for the ocular manifestations of IgAN. In our case report, scleral inflammation was only reduced 40 days after three consecutive daily pulse therapy sessions with methylprednisolone followed by an oral corticosteroid-tapering regimen. Furthermore, considering the severity of inflammation, the inherent risk of new episodes of scleritis, and the poor response to intravenous corticotherapy, the introduction of a stronger immunosuppressive agent is recommended for a long disease remission^(13)^. Further studies are warranted to evaluate the ocular involvement in Berger’s disease and whether IgAN-related scleritis is associated with a poor response to treatment with immunomodulators.
